# Effect of high-dose melphalan on marrow and intestinal epithelium in mice pretreated with cyclophosphamide.

**DOI:** 10.1038/bjc.1978.173

**Published:** 1978-07

**Authors:** J. L. Millar, B. N. Hudspith, T. J. McElwain, T. A. Phelps

## Abstract

The lethal effect of high-dose melphalan in mice could be offset by pretreatment with cyclophosphamide, cytosine arabinoside or low-dose melphalan. The reason for improved survival is unclear. Althoug animals given high-dose melphalan died with symptoms of gut death, in only one instance, that with low-dose melphalan itself, did pretreatment protect the intestinal epithelium as measured by the microcolony assay. A small enhancement in the recovery of the haemopoietic tissue in pretreated animals was noted, although this on its own is unlikely to explain the phenomenon. Experiments in tumour-bearing mice showed that pretreatment with cyclophosphamide did not reduce the toxicity of melphalan to the Lewis lung carcinoma.


*Br. J. Cancer* (1978) **38**, 137

### EFFECT OF HIGH-DOSE MELPHALAN ON MARROW AND INTESTINAL EPITHELIUM IN MICE PRETREATED WITH CYCLOPHOSPHAMIDE

J. L. MILLAR*, B. N. HUDSPITH*, T. J. McELWAIN† AND T. A. PHELPS*

*From the *Division of Biophysics and the †Division of Medicine, Institute of Cancer Research and Royal Marsden Hospital, Sutton, Surrey SM2 5PT*

Received 3 January 1978 Accepted 24 April 1978

**Summary.—The lethal effect of high-dose melphalan in mice could be offset by pretreatment with cyclophosphamide, cytosine arabinoside or low-dose melphalan. The reason for improved survival is unclear. Although animals given high-dose melphalan died with symptoms of gut death, in only one instance, that with low-dose melphalan itself, did pretreatment protect the intestinal epithelium as measured by the microcolony assay. A small enhancement in the recovery of the haemopoietic tissue in pretreated animals was noted, although this on its own is unlikely to explain the phenomenon. Experiments in tumour-bearing mice showed that pretreatment with cyclophosphamide did not reduce the toxicity of melphalan to the Lewis lung carcinoma.**

SEVERAL binary combinations of cytotoxic agents have been reported to have less than the expected toxicity on normal tissues in mice and rats. These combinations generally take the form of a small dose (the pretreatment or priming dose) followed by a large challenge dose of the same (Jeney *et al.*, 1968; Rose *et al.*, 1975; Millar and McElwain, 1978) or different cytotoxic agent (Smith and Wilson, 1967; Jeney *et al.*, 1968; Schmidt *et al.*, 1970; Millar *et al.*, 1975; Millar and Hudspith 1976; Millar *et al.*, 1978).

In this communication, the effect of various pretreatments in reducing the toxicity of a large dose of melphalan (Melph) in mice is reported. The reaction of the clonogenic cells in marrow and intestinal epithelium to a combination of cyclophosphamide (Cyclo) and Melph is investigated. Bearing in mind the importance of normal-tissue-sparing combinations in cancer chemotherapy, the effectiveness of the sparing combination of Cyclo and Melph on a murine tumour, the Lewis lung carcinoma is also reported.

### MATERIALS AND METHODS

*Animals.*—8–10-week-old CBA or C57BL male mice were used in survival studies, with never less than 10 animals per point. CBA mice were used in normal-tissue toxicity studies, whereas C57BL mice were used in experiments involving the Lewis lung carcinoma. Mice ranged in weight between 22 and 27 g at the start.

*Drugs.*—All drugs were administered i.p. Pure cyclophosphamide monohydrate (Cyclo) (Endoxana, Koch-Light Ltd) was used. It was dissolved in saline, as was cytosine arabinoside. (Ara C) (Cytosar, Upjohn Ltd). Melphalan (Melph) (Alkeran, Burroughs Wellcome & Co.) was first dissolved in 1 ml of 2% (v/v) acid alcohol and then in saline to the required concentrations. All solutions were prepared shortly before use.

*Haemopoietic stem-cell assay.*—Stem cells of the femoral marrow were assayed using the technique of Till and McCulloch (1961). The procedure for measuring peripheral-blood leucocyte and granulocyte levels has been reported elsewhere (Millar and Hudspith, 1976). There were 5 donor animals per group and never less than 8 animals in recipient groups or groups used to study peripheral leucocyte recovery.

*Studies on the intestinal epithelium.*—Crypt regeneration in the jejunum after Melph treatment was assessed by a method similar to the microcolony technique described by Withers and Elkind (1970) after high doses of radiation. Mice were either treated with 20 mg/kg Melph alone, or treated on various days before with either 50 mg/kg Cyclo, 200 mg/kg Ara C or 5 mg/kg Melph, followed by 20 mg/kg Melph. Four days after the high Melph injection, mice were given 20 $\mu$Ci $^3$H-TdR (Radiochemical Centre, Amersham) each and killed 40 min later. Three portions of each jejunum were fixed in 10% formalin and then cut into 4–5 $\mu$m sections. Autoradiography was carried out to facilitate identification of viable regenerating crypts. Sections were stained with haematoxylin and eosin and counts made of regenerating crypts/circumference and expressed as a proportion of the number of crypts/circumference in untreated mice.

*Lewis lung tumour.*—Lewis lung tumours were grown i.m. and treated *in situ* with Melph. Some groups of mice received 50 mg/kg Cyclo 2 days before the Melph. Either 18 h or 72 h after the Melph treatment, single-cell suspensions were made from the tumours, and the number of surviving clonogenic cells was assessed using the lung colony assay (Steel and Adams, 1975). The lung colonies were derived from surviving clonogenic tumour cells, and the fraction of surviving clonogenic cells per tumour was calculated, as has previously been described (Stephens and Peacock, 1977).

FIG. 1.—Survival of animals given (a) 50 mg/kg Cyclo at various times before 20 mg/kg (●), 30 mg/kg (▲) or 40 mg/kg (■) Melph; (b) 200 mg/kg Ara C at various times before 20 mg/kg (●), 30 mg/kg (▲) or 40 mg/kg (■) Melph; (c) 5 mg/kg Melph at various times before 20 mg/kg (●) or 25 mg/kg (■) Melph. The effects of the various challenge doses on their own are shown on the right of each figure (±s.e.).

### RESULTS

### *Survival experiments*

A dose of 20 mg/kg Melph killed 9/10, 8/10 and 7/10 animals in 3 separate experiments. Thus, on average this dose of Melph killed 8/10 animals (Figs. 1a, b, c) 5–6 days after treatment. Higher doses, 25 mg/kg, 30 mg/kg and 40 mg/kg proved fatal to all the animals in 5 days or less. A dose of 50 mg/kg Cyclo was given at various times before 20, 30 or 40 mg/kg Melph (Fig. 1a). Improved survival was seen for at least some intervals, at each dose level. At the lowest dose of Melph greatest survival was seen when there was an interval of 2–3 days between pretreatment and challenge dose. At the higher doses of Melph, although few animals survived, an interval of 1 or 2 days seemed more effective.

Ara C is effective as a pretreatment for Melph (Fig. 1b). A dose of 200 mg/kg Ara C offset the lethal effect of 20 mg/kg Melph, and increased survival after 30 mg/kg Melph, when the interval between the administrations was 1 or 2 days. This pretreatment with Ara C did not improve survival after 40 mg/kg Melph.

A pretreatment dose of 5 mg/kg Melph 2 or 3 days before 20 mg/kg Melph increased the survival from 20% to 100% (Fig. 1c). When the challenge dose was

FIG. 2.—Microcolonies in the gut 4 days after 20 mg/kg Melph, in animals pretreated with 50 mg/kg Cyclo (C) 200 mg/kg Ara C (A) or 5 mg/kg Melph (M). C3M indicates that Cyclo was given 3 days before Melph, etc. Mean ± s.e.

FIG. 3.—Survival of marrow stem cells after various doses of Melph. Normal animals (●), animals given 50 mg/kg Cyclo 2 days before (○). Mean ± s.e.

increased to 25 mg/kg, 5 mg/kg Melph treatment 2 days before the larger dose improved survival.

*Effect of melphalan on the intestinal epithelium*

The average number of regenerating crypts per circumference of the jejunum of mice 4 days after 20 mg/kg Melph is shown in Fig. 2. All these colonies labelled with $^3$H-TdR, suggesting that they were viable colonies acting as foci for the regeneration of the intestinal epithelium. The crypt survival of animals given 50 mg/kg Cyclo 3, 2 or 1 day before 20 mg/kg Melph shows that animal survival (Fig. 1a) may not have been related to crypt survival with this treatment. Nor did the administration of 200 mg/kg Ara C 2 days before 20 mg/kg Melph improve crypt survival, though this treatment did not improve animal survival either (Fig. 1b). With Melph pretreatment, there was a correlation between crypt survival and animal survival (Fig. 1c).

*Survival of marrow stem cells after melphalan*

The survival of marrow stem cells (CFU-S) after various doses of Melph in normal animals and in animals given 50 mg/kg Cyclo 2 days before, is shown in Fig. 3. There is no evidence that pretreatment with Cyclo altered the sensitivity of the stem cells to Melph. The small vertical separation between the lines indicates the effect of the 50 mg/kg Cyclo pretreatment.

*Recovery of marrow stem cells after melphalan*

The recovery of bone marrow stem cells after 15 mg/kg Melph was followed in otherwise normal animals and in animals given 50 mg/kg Cyclo 2 days before (Fig. 4). This dose of Melph allowed all control animals to survive the duration of the experiment. It can be seen that, although the depression is slightly greater in animals that received the pretreatment, recovery took place at a greater rate. At Day 6, for instance, the number of stem cells in pre-

FIG. 4.—Recovery of marrow stem cells after 15 mg/kg Melph. Normal animals, solid symbols; animals given 50 mg/kg Cyclo 2 days before the Melph, open symbols. Mean ±s.e.

treated animals was back to normal, whereas in animals that received no pretreatment they were only 10% of normal.

*Recovery of peripheral-blood leucocytes after melphalan*

The peripheral-blood leucocyte level was initially lower in animals given 50 mg/kg Cyclo 2 days before 15 mg/kg Melph than in animals that only received 15 mg/kg Melph (Fig. 5). By Day 6, however, there were 4 times as many peripheral leucocytes in pretreated animals as in animals that received Melph alone. Also, in pretreated animals there was an overshoot between Days 8 and 22 which was not seen in animals that received Melph alone. It is noteworthy that the proportion of granulocytes in the leucocyte count increased steadily in both pretreated and control animals during the course of the recovery.

FIG. 5.—Recovery of peripheral leucocytes (circles) and granulocytes (squares) in animals given 15 mg/kg Melph (solid symbols) or 50 mg/kg Cyclo 2 days before 15 mg/kg Melph (open symbols). Mean±s.e.

FIG. 6.—Effect of various doses of Melph on survival of clonogenic cells (SF=surviving fraction) in tumours in normal animals (solid symbols) or in animals treated with 50 mg/kg Cyclo 2 days before (open symbols). The assay was performed 18 h (solid lines) or 72 h (broken lines) after the melphalan. Different shaped symbols refer to separate experiments.

*Effect of melphalan on clonogenic Lewis lung tumour cells*

Fig. 6 shows that Lewis lung tumour cells were sensitive to Melph over the dose range used for the normal tissue studies. The sensitivity of these clonogenic tumour cells was not altered if the animals were treated with 50 mg/kg Cyclo 2 days before the Melph. The small vertical displacements between the lines shows the effect of 50 mg/kg Cyclo on clonogenic tumour-cell survival. The assay was performed at 18 h and at 3 days after the Melph challenge.

### DISCUSSION

There are a number of instances in which it has been shown that pretreatment with a cytotoxic agent increases the tolerance of an animal to a subsequent large dose of the same or a different cytotoxic agent.

In work with radiation, with challenge doses up to 1000 rad when marrow is the critical tissue, we have noted that the response of marrow stem cells was the same in normal and pretreated animals, and that recovery, when it got under way in each situation, proceeded at the same rate (Millar *et al.*, 1978). The difference was in the time at which recovery began. In pretreated animals there was little or no post-irradiation lag, recovery beginning promptly after irradiation (Smith *et al.*, 1968; Hanks and Ainsworth, 1967; Brecher *et al.*, 1967; Millar *et al.*, 1978). This suggests that the pretreatment dose prepared the system for the challenge dose, and recovery began promptly after the challenge dose.

At higher doses of radiation the intestinal epithelium becomes the critical tissue. Pretreatment with Ara C 12 h before irradiation reduces the toxicity of the radiation on this tissue (Boarder, 1976). In this situation there appears to be a reduction in the radiosensitivity of the tissue.

Pretreatment with a small dose of Cyclo will offset lethality of an otherwise lethal Cyclo dose (Millar and McElwain, 1978). In this instance neither the marrow nor the intestinal epithelium differed in their response or recovery after Cyclo and the cause of death, and therefore the mechanism by which pretreatment operated could not be established. It was noted, however, that the urothelium in pretreated animals was in much better condition 24 h after a large dose of Cyclo, if the animals had been pretreated with the same drug.

In the present communication we have demonstrated that pretreatment with Cyclo will reduce the toxicity of Melph in mice. Although animals treated with Melph alone appeared to die from gut failure (death after about 5 days, with diarrhoea and gross histological changes in the intestinal epithelium) it could not be established that the sparing pretreatment with Cyclo improved the condition of the intestinal epithelium, at least, using the microcolony test available for study of this tissue. The mechanism for improved survival, therefore, remains unclear. It was established that Cyclo pretreatment did not alter the sensitivity of marrow stem cells to a subsequent challenge of graded doses of Melph up to and including 20 mg/kg, yet there was a slightly earlier recovery of these stem cells in animals given 15 mg/kg Melph and the Cyclo pretreatment. It is of interest that the peripheral leucocyte count also recovered more rapidly in these animals.

It is unlikely that the enhanced recovery of the stem cells of the marrow could have led to the quicker recovery in the peripheral blood, because of the extremely short interval between recovery of stem cells and the onset of peripheral leucocyte recovery. Previous work (Millar *et al.*, 1978) in which enhanced survival of marrow improved the survival of irradiated mice, has shown that an interval of about 8 days is required for improved survival of stem cells to be reflected in increased peripheral leucocyte number.

Although the results of the survival experiments could not be established with great accuracy, as large numbers of animals were not used, it appeared from Fig. 1 that, as a rule, pretreatment 2 days before a challenge with high-dose Melph provided the best survival, and this was the case whether Cyclo, Ara C or low-dose

Melph was used as the pretreatment. This is consistent with the timing where radiation was used as the challenge, and various cytotoxic agents offset lethality (Millar *et al.*, 1978).

In conclusion, a similar type of recovery to that studied in the marrow (Millar *et al.*, 1978) in pretreated animals cannot be shown to operate in the gut by our present methods. Other mechanisms, which might involve interaction between gut and marrow, must remain speculative. It is noteworthy that Lewis lung tumour tissue was *not* spared by Cyclo pretreatment, and the potential therapeutic advantage of pretreatment before large-dose challenge should therefore be stressed. Pilot studies in the clinic are in progress and have so far yielded encouraging results.

The authors with to express their thanks to Dr Gordon Steel for helpful discussion and advice and to Miss Kay Adams for her expert technical assistance.

REFERENCES

BOARDER, T. A. (1976) The effect of cytotoxic agents on intestinal stem cells. Ph.D. Thesis. Univ. of London. p. 178.

BRECHER, G., SMITH, W. W., WILSON, S. & FRED, S. (1967) Kinetics of colchicine-induced hemopoietic recovery in irradiated mice. *Radiat. Res.*, **30**, 600.

HANKS, G. E. & AINSWORTH, E. J. (1967) Endotoxin protection and colony-forming units. *Radiat. Res.*, **32**, 367.

JENEY, A. JR., CONNORS, T. A. & JONES, M. (1968) The toxicity of merophan after pretreatment with subtoxic dose. *Acta Physiol. Acad. Sci. Hung.*, **33**, 89.

MILLAR, J. L., BLACKETT, N. M. & HUDSPITH, B. N. (1978) Enhanced post-irradiation recovery of the haemopoietic system in animals pretreated with a variety of cytotoxic agents. *Cell Tissue Kinet.*, **11**, 543.

MILLAR, J. L. & HUDSPITH, B. N. (1976) Sparing effect of cyclophosphamide (NSC-26271) pretreatment on animals lethally treated with $\gamma$-irradiation. *Cancer Treat. Rep.*, **60**, 409.

MILLAR, J. L., HUDSPITH, B. N. & BLACKETT, N. M. (1975) Reduced lethality in mice receiving a combined dose of cyclophosphamide and busulphan. *Br. J. Cancer*, **32**, 193.

MILLAR, J. L. & MCELWAIN, T. J. (1978) Combinations of cytotoxic agents that have less than expected toxicity on normal tissues in mice. In *Fundamentals in cancer chemotherapy. Antibiot. Chemother.*, **23**, 271.

ROSE, W. C., RIMM, A. A., SALTZSTEIN, E. C., TRUITT, R. L. & BORTIN, M. M. (1975) Low-dose chemotherapy as a prelude to intensive treatment of spontaneous leukaemia-lymphoma in AKR mice. *J. Natl. Cancer Inst.*, **55**, 219.

SCHMIDT, L. H., MONTGOMERY, J. A., LASTER, W. R. JR. & SCHABEL, F. M. JR. (1970) Combination therapy with arabinosyl cytosine and thioguanine. *Proc. Am. Ass. Cancer Res.*, **11**, 70.

SMITH, W. W. & WILSON, S. M. (1967) Effect of vinblastine and vincristine on survival and hemopoiesis in irradiated mice. *J. Natl. Cancer Inst.*, **39**, 1055.

SMITH, W. W., WILSON, S. M. & FRED, S. S. (1968) Kinetics of stem cell depletion and proliferation: Effects of vinblastine and vincristine in normal and irradiated mice. *J. Natl. Cancer Inst.*, **40**, 847.

STEEL, G. G. & ADAMS, K. (1975) Stem-cell survival and tumour control in the Lewis lung carcinoma. *Cancer Res.*, **35**, 1530.

STEPHENS, T. C. & PEACOCK, J. H. (1977) Tumour volume response, initial cell kill and cellular repopulation in B16 melanoma treated with cyclophosphamide and 1-(2-chloroethyl)-3-cyclohexyl-1-nitrosourea. *Br. J. Cancer*, **36**, 313.

TILL, J. E. & MCCULLOCH, E. A. (1961) A direct measurement of the radiation sensitivity of normal mouse bone marrow cells. *Radiat. Res.*, **14**, 213.

WITHERS, H. R. & ELKIND, M. M. (1970) Microcolony survival assay for cells of the mouse intestinal mucosa exposed to radiation. *Int. J. Radiat. Biol.*, **17**, 261.


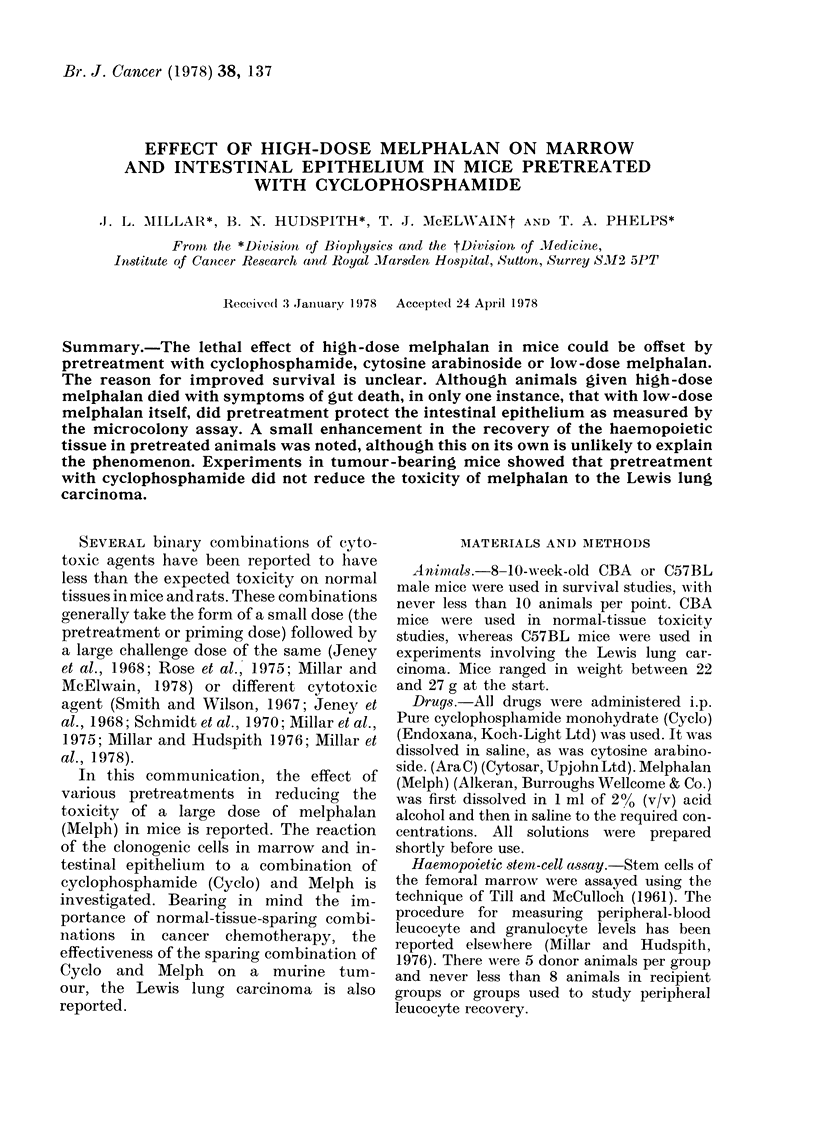

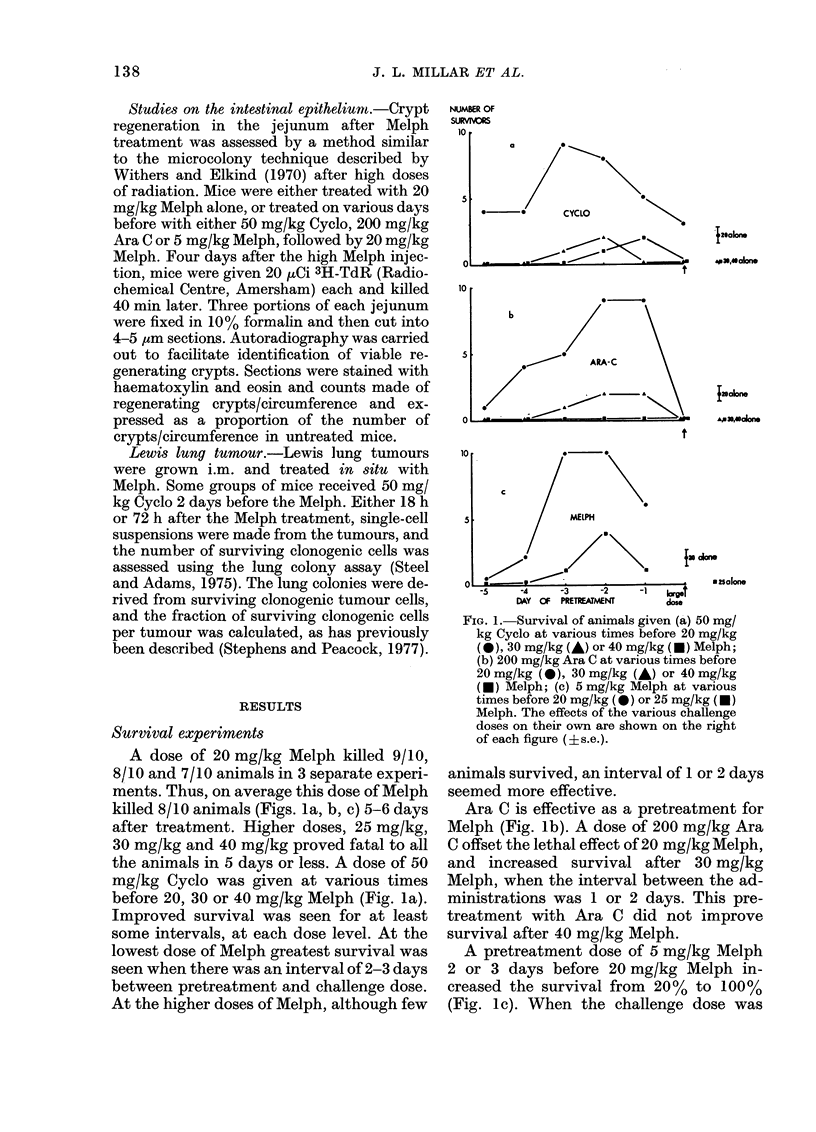

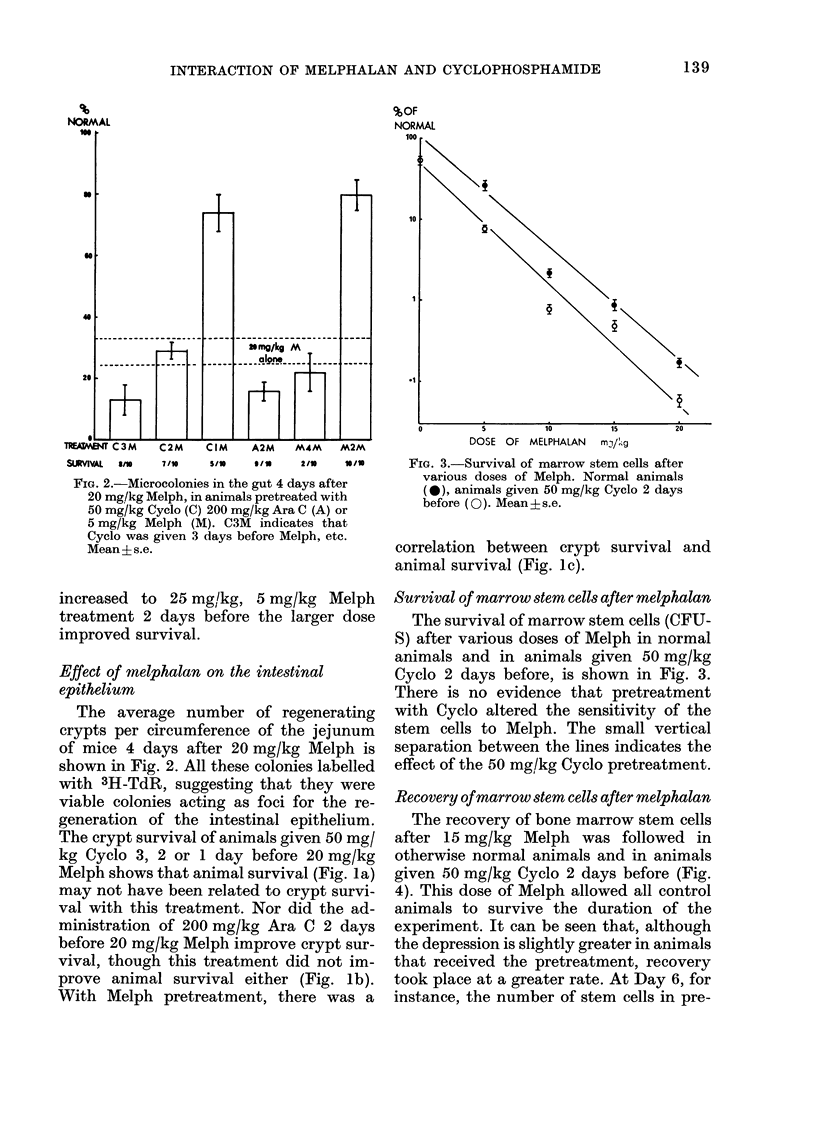

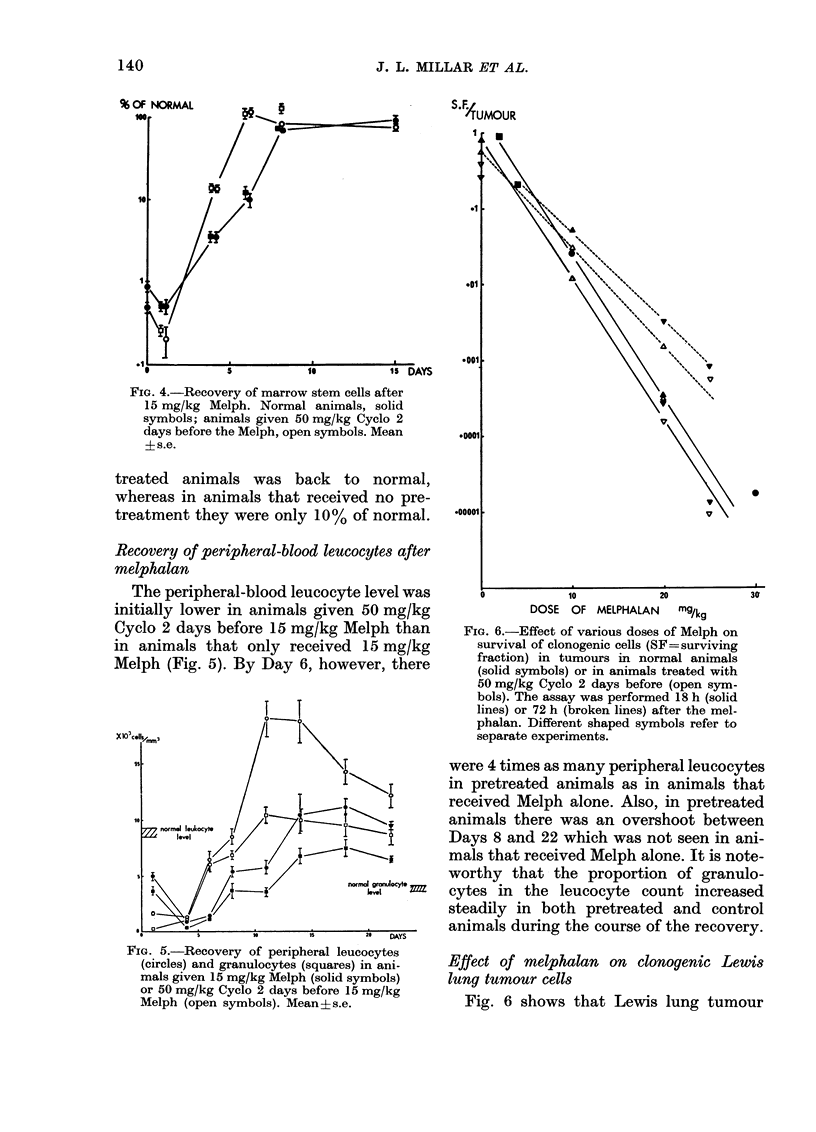

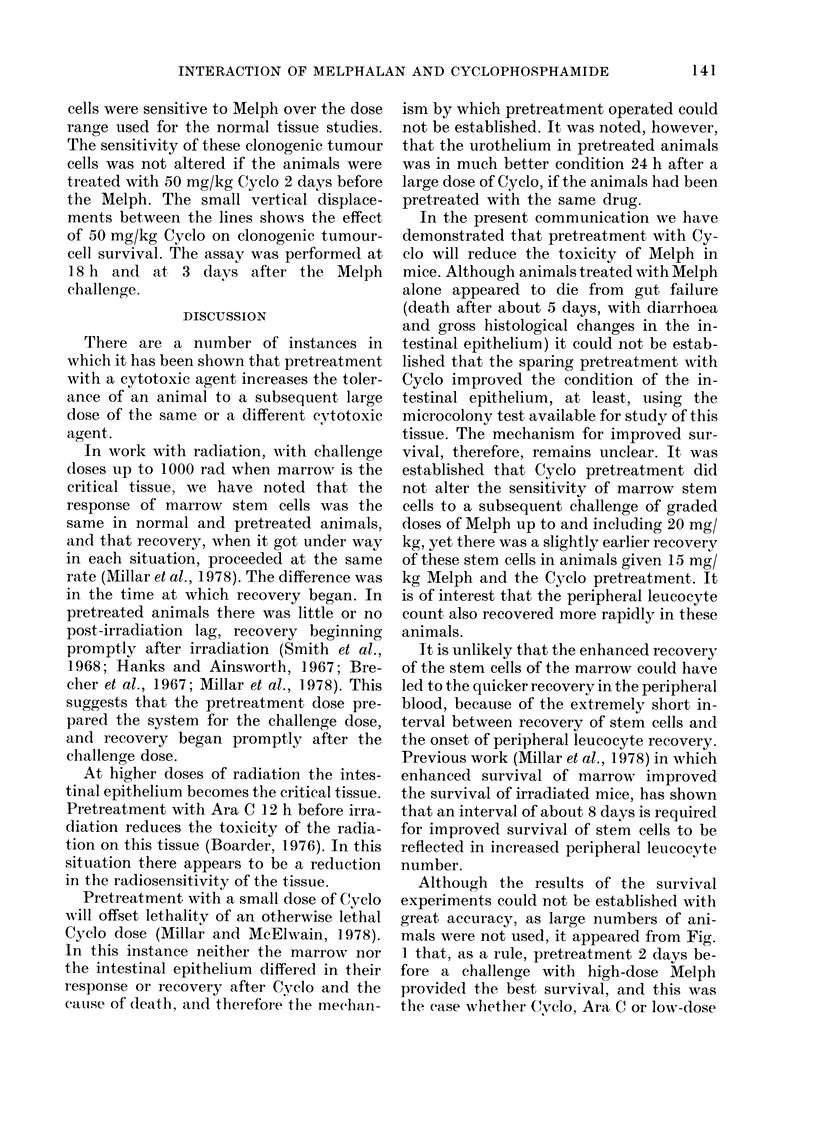

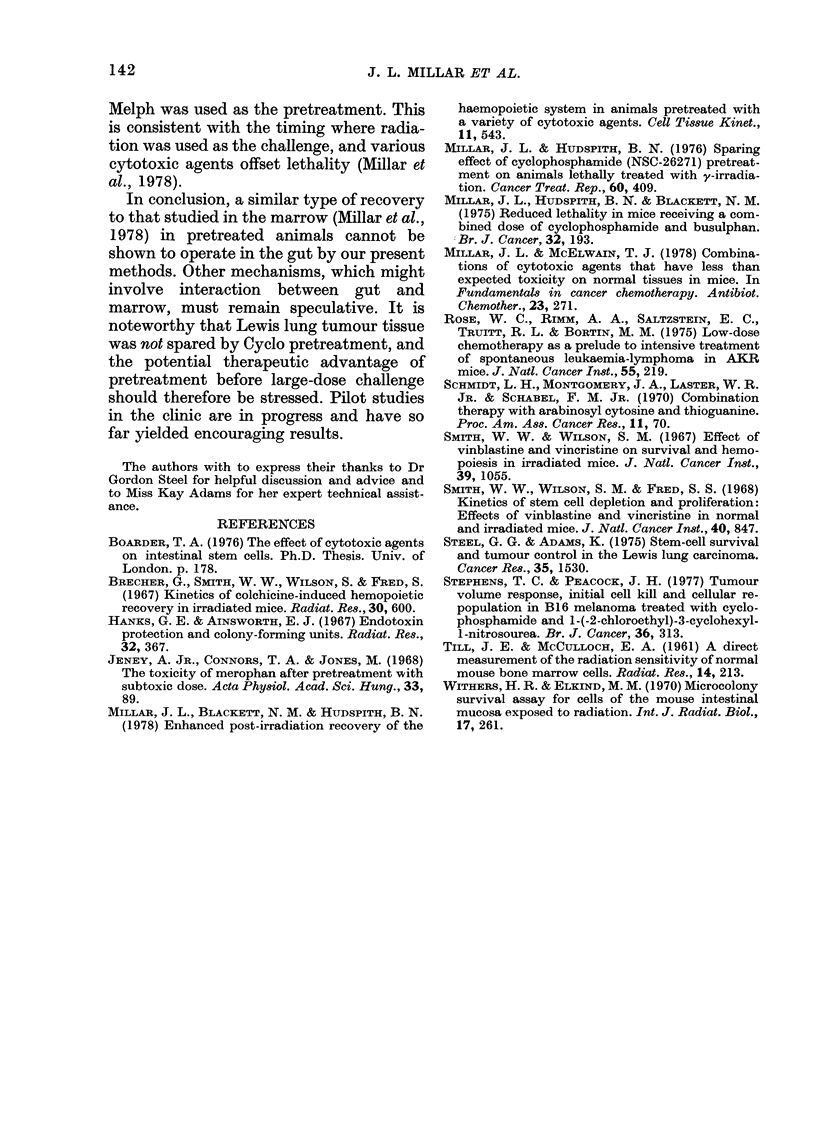


## References

[OCR_00553] Brecher G., Smith W. W., Wilson S., Fred S. (1967). Kinetics of colchicine-induced hemopoietic recovery in irradiated mice.. Radiat Res.

[OCR_00558] Hanks G. E., Ainsworth E. J. (1967). Endotoxin protection and colony-forming units.. Radiat Res.

[OCR_00563] Jeney A., Connors T. A., Jones M. (1968). The toxicity of merophan after pretreatment with subtoxic dose.. Acta Physiol Acad Sci Hung.

[OCR_00569] Millar J. L., Blackett N. M., Hudspith B. N. (1978). Enhanced post-irradiation recovery of the haemopoietic system in animals pretreated with a variety of cytotoxic agents.. Cell Tissue Kinet.

[OCR_00583] Millar J. L., Hudspith B. N., Blackett N. M. (1975). Reduced lethality in mice receiving a combined dose of cyclophosphamide and busulphan.. Br J Cancer.

[OCR_00577] Millar J. L., Hudspith B. N. (1976). Sparing effect of cyclophosphamide (NSC-26271) pretreatment on animals lethally treated with gamma-irradiation.. Cancer Treat Rep.

[OCR_00589] Millar J. L., McElwain T. J. (1978). Combinations of cytotoxic agents that have less than expected toxicity on normal tissues in mice.. Antibiot Chemother (1971).

[OCR_00596] Rose W. C., Rimm A. A., Saltzstein E. C., Truitt R. L., Bortin M. M. (1975). Low-dose chemotherapy as a prelude to intensive treatment of spontaneous leukemia-lymphoma in AKR mice.. J Natl Cancer Inst.

[OCR_00609] Smith W. W., Wilson S. M. (1967). Effects of vinblastine and vincristine on survival and hemopoiesis in irradiated mice.. J Natl Cancer Inst.

[OCR_00615] Smith W. W., Wilson S. M., Fred S. S. (1968). Kinetics of stem cell depletion and proliferation: effects of vinblastine and vincristine in normal and irradiated mice.. J Natl Cancer Inst.

[OCR_00620] Steel G. G., Adams K. (1975). Stem-cell survival and tumor control in the Lewis lung carcinoma.. Cancer Res.

[OCR_00625] Stephens T. C., Peacock J. H. (1977). Tumour volume response, initial cell kill and cellular repopulation in B16 melanoma treated with cyclophosphamide and 1-(2-chloroethyl)-3-cyclohexyl-1-nitrosourea.. Br J Cancer.

[OCR_00632] TILL J. E., McCULLOCH E. A. (1961). A direct measurement of the radiation sensitivity of normal mouse bone marrow cells.. Radiat Res.

[OCR_00637] Withers H. R., Elkind M. M. (1970). Microcolony survival assay for cells of mouse intestinal mucosa exposed to radiation.. Int J Radiat Biol Relat Stud Phys Chem Med.

